# Human Monoclonal Antibody Targeting the Heparan Sulfate Chains of Glypican-3 Inhibits HGF-Mediated Migration and Motility of Hepatocellular Carcinoma Cells

**DOI:** 10.1371/journal.pone.0137664

**Published:** 2015-09-02

**Authors:** Wei Gao, Heungnam Kim, Mitchell Ho

**Affiliations:** Antibody Therapy Section, Laboratory of Molecular Biology, Center for Cancer Research, National Cancer Institute, National Institutes of Health, Bethesda, MD, 20892, United States of America; University of Hong Kong, HONG KONG

## Abstract

Heparan sulfate proteoglycans (HSPGs) participate in many processes related to tumor development, including tumorigenesis and metastasis. HSPGs contain one or more heparan sulfate (HS) chains that are covalently linked to a core protein. Glypican-3 (GPC3) is a cell surface-associated HSPG that is highly expressed in hepatocellular carcinoma (HCC). GPC3 is involved in Wnt3a-dependent HCC cell proliferation. Our previous study reported that HS20, a human monoclonal antibody targeting the HS chains on GPC3, inhibited Wnt3a/β-catenin activation. In the current study, we showed that the HS chains of GPC3 could mediate HCC cells’ migration and motility. Knocking down GPC3 or targeting the HS chains by HS20 inhibited HCC cell migration and motility. However, HS20 had no effect on GPC3 knockdown cells or GPC3 negative cells. In addition, an antibody that recognizes the core protein of GPC3 did not change the rate of cell motility. HCC cell migration and motility did not respond to either canonical or non-canonical Wnt induction, but did increase under hepatocyte growth factor (HGF) treatment. HS20-treated HCC cells exhibited less ability for HGF-mediated migration and motility. Furthermore, HS20 inhibited *in vitro* HCC spheroid formation and liver tumor growth in mice. GPC3 interacted with HGF; however, a mutant GPC3 lacking the HS chain showed less interaction with HGF. Blocking the HS chains on GPC3 with HS20 reduced c-Met activation in HGF-treated HCC cells and 3D-cultured spheroids. Taken together, our study suggests that GPC3 is involved in HCC cell migration and motility through HS chain-mediated cooperation with the HGF/Met pathway, showing how HS targeting has potential therapeutic implications for liver cancer.

## Introduction

Hepatocellular carcinoma (HCC) accounts for 70% of liver malignancies, making it the fifth most common and the third most lethal malignancy in the world [1]. Only a small proportion of HCCs diagnosed at an early stage have treatment options. Most HCC cases are identified at an advanced stage, when resistance to most chemotherapeutic drugs is profound. In general, the survival rate is low and surgery is the most viable treatment option [2,3]. Therefore, the development of effective therapeutic approaches to treat HCC is urgently needed.

Heparan sulfate proteoglycans (HSPGs) characteristically have a core protein with one or more heparan sulfate (HS) chains [4]. HSPGs function as cell surface co-receptors by interacting with extracellular molecules, including growth factors, chemokines, and cell-extracellular matrix (ECM) proteins to influence cell growth, differentiation, and tumorigenicity [5]. Glypican-3 (GPC3) is a HSPG that is specifically expressed in HCC [6]. As an oncofetal antigen, GPC3 is highly expressed in over 70% of HCCs but not in normal adult tissues [7]. The expression of GPC3 is correlated with poor clinical prognosis for HCC survival [8]. GPC3 knockdown has been shown to slow tumor growth in mice [9]. There is also evidence that shows that GPC3 promotes HCC proliferation by regulating Wnt and Yap signaling [10,11]. We generated HS20, a HS-specific antibody targeting GPC3, and found that HS20 inhibited HCC tumor growth by blocking canonical Wnt-signaling. However, HS20 also showed anti-tumor activity on cells with a β-catenin mutation [9], suggesting other mechanisms by which HS is involved.

The hepatocyte growth factor (HGF)/Met pathway is critical for liver development [12]. HGF and its receptor Met protect the liver from injury and damage by providing pivotal survival and anti-apoptotic signals [13–15]. Studies show that *HGF* or *Met* knockout mice have impaired development of embryonic liver [16,17]. In HCC, various components of the HGF/Met pathway are reported to contribute to HCC progression [18,19]. Gene signature analysis indicates that 40% of HCC patients show Met activation and poor prognosis [20]. Therapeutic candidates that target the HGF/Met pathway by monoclonal antibodies or small molecules are currently under clinical evaluation. Most of the potential candidates are still at an early stage [12,21]. Emerging evidence demonstrates that HSPGs interact with HGF through HS moieties in order to promote HGF-mediated signaling and subsequently tumor pathogenesis. Disruption of HS function on HSPGs causes the loss of HGF function and affects morphogenesis and tumorigenicity [22–24].

We showed that the HS chains of GPC3 are important for HGF binding and c-Met activation. Blocking the HS chains by HS20 inhibited HGF-induced HCC cell migration, motility, and 3D-spheroid formation. In conclusion, our study suggests that GPC3 is involved in tumor cell motility via HS chain-mediated coordination with the HGF/Met pathway. Targeting the HS chains of GPC3 could inhibit HCC tumor pathogenesis through multiple mechanisms.

## Materials and Methods

### Cell lines, recombinant protein

Hep3B and HepG2 cell lines were obtained from the American Type Culture Collection (ATCC, Manassas, VA). The Huh-7 [25] and SK-hep1 cell line (ATCC, Manassas, VA) were obtained from Xin Wei Wang in the NCI Laboratory of Human Carcinogenesis. Cell lines were cultured in DMEM (Invitrogen, Camarillo, CA), supplemented with 10% fetal bovine serum (FBS) (Thermo Scientific, Asheville, NC), 100 U/mL penicillin, 0.1 mg/mL streptomycin, and 2 mmol/L L-glutamine. Recombinant GPC3-hFc, GPC3ΔHS-hFc, and CD22-hFc were purified as we described earlier [11,26]. Hep3B knockdown cells were constructed by using GPC3 gene-specific sh-RNA as described in our previous work [11]. HGF knockdowns were performed using SMART-POOL siRNA from Dharmacon/GE Healthcare (Lafayette, CO).

### Western blotting and antibodies

Cells were seeded into a 6-well plate at 0.5 million/well. When they grew to 70–80% confluence, cells were starved with DMEM containing 1% FBS for 24 hours. The cells were then lysed or treated with 50 ng/mL HGF for 10 minutes. In some cases, cells were pre-treated with 50μg/mL HS20 or human IgG (Sigma, St. Louis, MO) for 30 minutes prior to HGF treatment. Cells were washed with cold PBS and then directly lysed with RIPA buffer (Cell Signaling Technology, Beverly, MA) for c-Met activation detection. The protein concentration of cell lysate was measured by a Coomassie blue assay (Pierce Biotechnology, Rockford, IL). Cell lysates (20 μg for each sample) were loaded into a 4–20% SDS-PAGE gel for electrophoresis. Antibodies against GPC3 were generated in our lab (YP7) [27]. Other antibodies were purchased: c-Met antibody, Santa Cruz Biotechnology (Dallas, TX, USA); phosphorylated c-Met antibody, Cell Signaling Technology (Beverly, MA); HGF antibody, R&D Systems Inc. (Minneapolis, MN); β-actin antibody, Sigma (St. Louis, MO). For c-Met activation in 3D-cultured tumor cells, spheroids were harvested by centrifugation, washed with cold PBS, and then directly lysed with RIPA buffer for c-Met activation detection.

### Wound healing assay

A culture insert (ibidi, Verona, WI) was put into each well of a 24-well plate, and 1x10^5^ cells were seeded into each sub-chamber. When the cells grew to 100% confluence, we removed the insert and filled the wells with 0.5 mL of growth medium. Cells were incubated at 37°C in a 5% CO_2_ incubator. Each well was examined and photographed by an AMG EVOS XL microscope (Advanced Microscopy Group, Bothell, WA) at indicated time points. For HGF knockdown, cells were first transfected with siRNA according to the manufacture’s protocol; after 60 hours, cells were seeded into wound healing inserts. In some cases, cells were pre-treated with 50 μg/mL HS20 or human IgG (Sigma St. Louis, MO) for 30 minutes before HGF treatment (50 ng/mL, R&D, Minneapolis, MN). These cells were also incubated at 37°C in a 5% CO_2_ incubator. Each well was then examined and photographed by an AMG EVOS XL microscope. The first image of each scratch was acquired at time zero using a phase contrast microscope at 10x magnification. The images were analyzed with the TScratch program [28].

### Cell motility assay

Motility assays were performed in 8 μm-pore transwells (Corning Inc. Life Science, Lowell, MA). Cells were cultured to 80% confluence and then starved for 24 h in DMEM containing 1% FBS. 5x10^4^ cells (or HGF siRNA transfected cells) were plated in 200μl of DMEM plus 1% FBS in the upper wells. Bottom wells contained 500μl of DMEM plus 1% FBS with or without HGF (100 ng/mL) (R&D, Minneapolis, MN). In some cases, both upper and bottom wells received 50 μg/mL HS20 or human IgG (Sigma St. Louis, MO). Cells were incubated at 37°C in humidified 5% CO2 for 16 h. Cells on the transwells were rinsed in PBS, fixed with 1% glutaraldehyde (Sigma, St. Louis, MO) in PBS at room temperature for 15 min, and stained with 0.1% crystal violet (Sigma St. Louis, MO) in water for 30 min. After de-staining in water, non-migrating cells on top of the filter were removed with a cotton swab. Each well was examined and photographed with an AMG EVOS XL microscope. Images were acquired through a phase contrast microscope at 10x magnification. Migrating cells on the bottom of the filter were solubilized in 500 μl of 0.2% Triton X-100 (Invitrogen, Camarillo, CA) at 4°C overnight. The absorbance was measured at 590 nm.

### Pull down assay

0.5 μg of recombinant human HGF (R&D, Minneapolis, MN) was mixed with 1 μg of GPC3-hFc, GPC3ΔHS-hFc, or CD22-hFc in 100 μl of RIPA buffer. The mixture was incubated on ice for 2 h. 100 μl of Agarose-Protein A beads (GE Healthcare, Pittsburgh, PA) were added into the mixture and rotated at 4°C for 2 h. The protein-Agarose beads complex was spun down and washed with RIPA buffer 5 times. The bound protein was released with 40 μl of 1x SDS loading buffer (Bio-rad Laboratories, Hercules, CA) for a Western blot.

### Tumor spheroid formation

Cells were seeded into a 6-well low attachment plate (Corning Inc. Life Science, Lowell, MA) at 5000 cells/well with DMEM supplemented with 10% FBS. 50 ng/mL recombinant human HGF (R&D, Minneapolis, MN), combined with HS20 or human IgG (50 μg/mL), was added to medium. The spheroid was photographed with a ZEISS HBO100 microscope on day 20. Images were acquired at 10x magnification. The volume of each spheroid is calculated by the formula v = 4πr^3^/3 (r represents the radius of spheroid).

#### Animal testing

All mice were housed and treated under the protocol approved by the Institutional Animal Care and Use Committee at the National Institutes of Health (NIH). 10 x 10^6^ Hep3B cells or 5 x 10^6^ two-round *in vivo* passaged HepG2 cells were suspended in 200 μl of PBS and inoculated subcutaneously into 4 to 6 week‐old female BALB/c nu/nu nude mice (NCI-Frederick Animal Production Area, Frederick, MD). Tumor dimensions were determined using calipers and tumor volume (mm^3^) and calculated with the formula V = ab^2^/2, where a and b represent tumor length and width, respectively. When the average tumor size reached approximately 100 mm^3^, mice were intravenously injected with 25 mg/kg HS20 (Hep3B tumor) or 20 mg/kg (HepG2 tumor) twice a week.

### Statistics

For all group data, experiments were repeated at least three times independently. All of the representative data was expressed as mean ± SD. Two-tailed Student’s t-tests were applied to determine significant differences, with P* <0.05 defined as significant. The GraphPad Prism 6 program (San Diego, CA) was used to statistically analyze the results.

## Results

### GPC3 regulated HCC cell migration and motility

**Fig 1:**
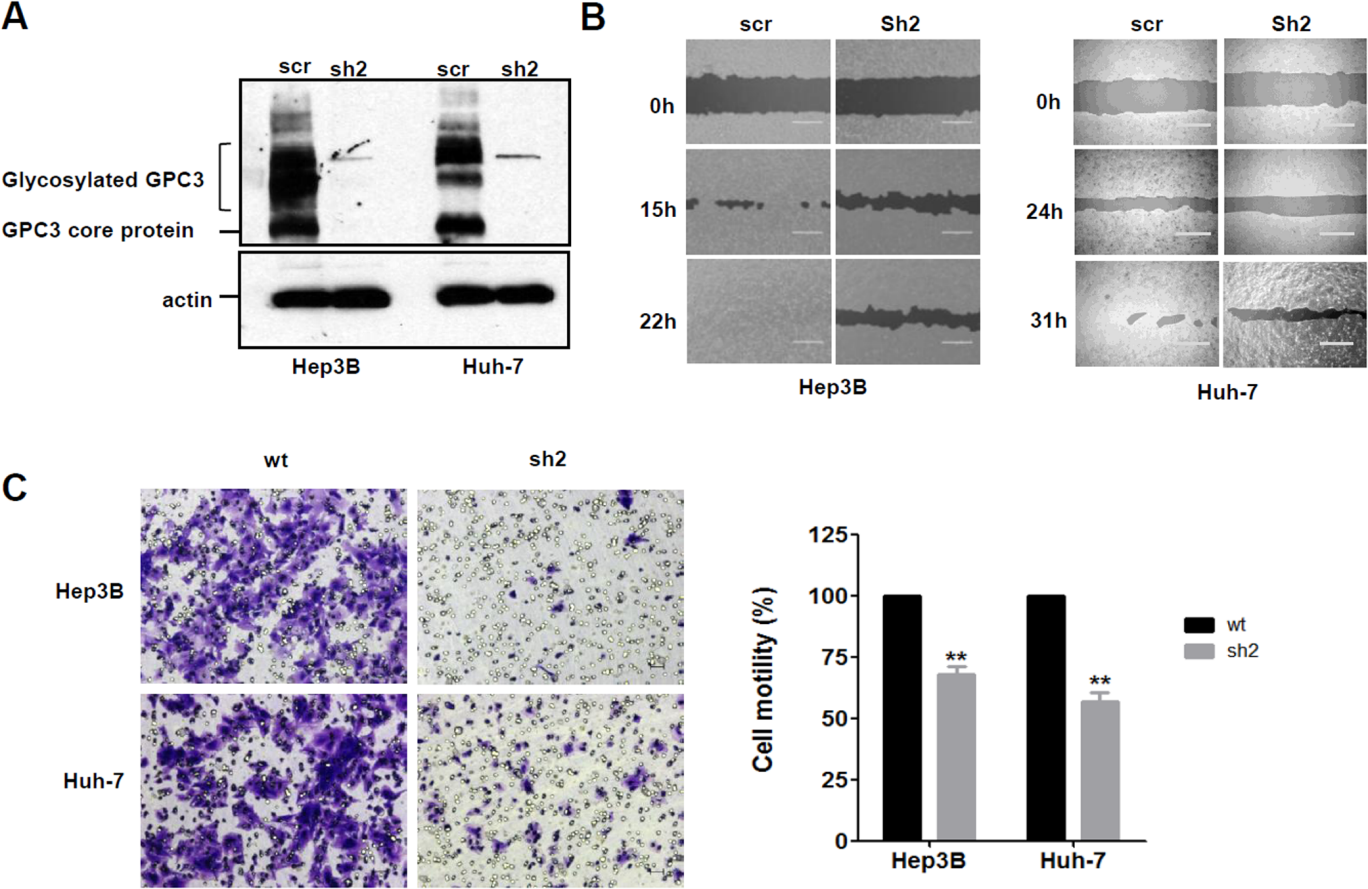
Knocking down GPC3 reduced cell migration and motility in HCC cells. (A) Western blot to show GPC3 knockdown efficiency in Hep3B cells and Huh-7 cells. (B) Wound healing assay to detect cell migration ability in GPC3 knocked down Hep3B cells and Huh-7 cells. Scale bar indicates 400 μm. (C) Trans-well assay to examine cell motility in wild type and GPC3 knocked down cells (sh2). Scale bar indicates 50 μm. The OD_590nm_ value of wild type group was set up as 100%. Values represent mean ± SD from three replicates. P**<0.01.

To investigate the function of GPC3 on cell motility, we established GPC3 knockdown cells lines [11] (Fig 1A). We compared the cell migration of GPC3 knockdown cells with that of control cells in wound healing assays. GPC3 knockdown cells had reduced migration ability compared to control cells in both Hep3B and Huh-7 cells (Fig 1B). In the cell motility assay, GPC3 knockdown cells had at least 30% less migration ability compared to wild type HCC cells (Fig 1C). However, knocking down GPC3 had no significant effect on cell invasion (data not shown), most likely because Hep3B and Huh-7 cells both belong to non-metastatic HCC cell lines. Overall, these observations suggest that GPC3 plays a role in HCC cell migration and motility.

### Blocking the HS chains of GPC3 inhibited HCC cell migration and motility

**Fig 2:**
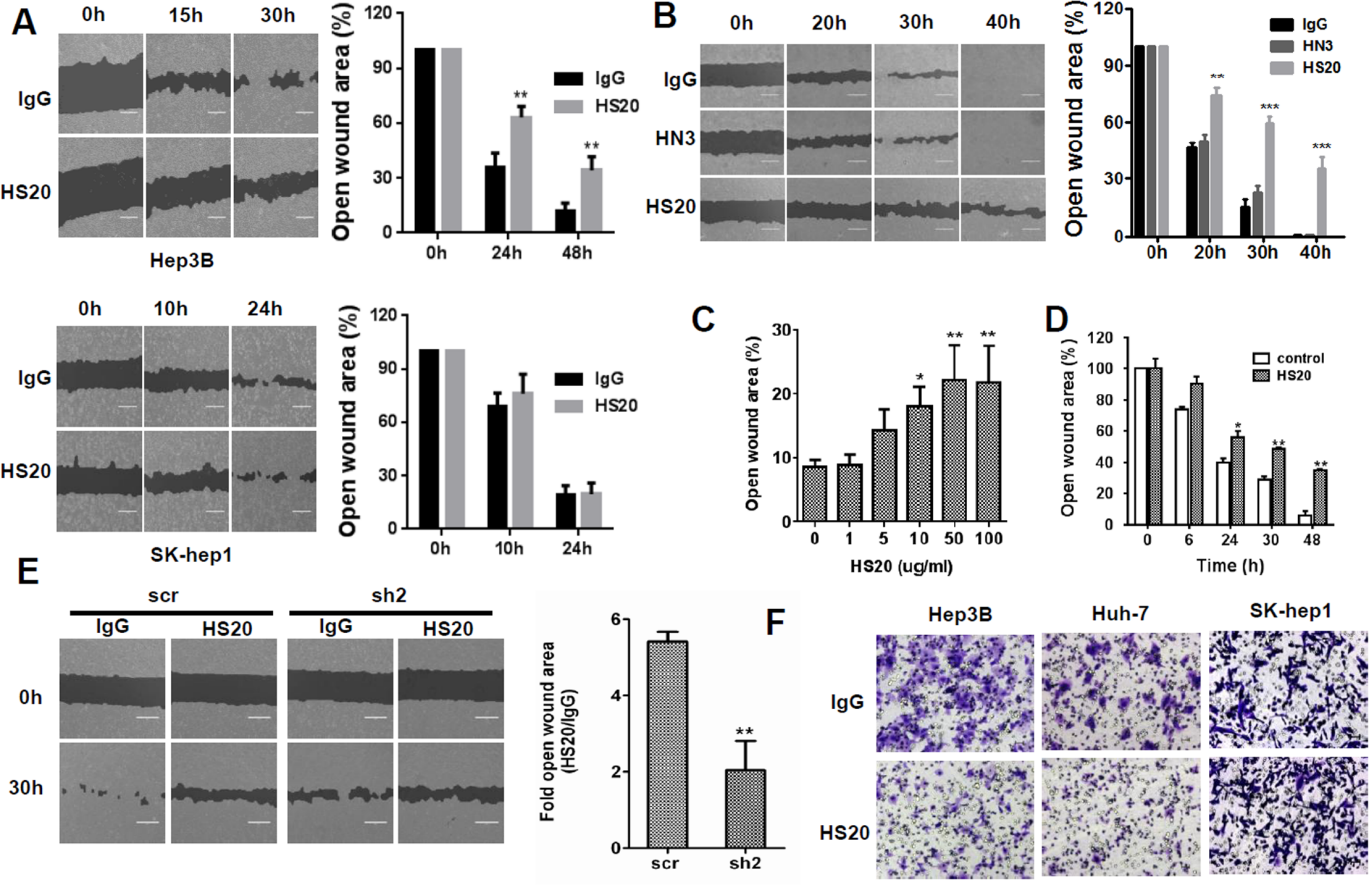
Blocking the HS chains of GPC3 by HS20 inhibited cell migration and motility in HCC cells. (A) Hep3B cells and SK-hep1 cells were treated with 50 μg/mL IgG or HS20. Cell migration ability was then measured in a wound healing assay. Scale bar indicates 400 μm. The open wound area at 0 hours was regarded as 100%. Values represent mean ± SD from three replicates. P**<0.01 compare to IgG group. (B) Hep3B cells were treated with 50 μg/mL of the indicated antibodies. Cell migration ability was then measured in a wound healing assay. Scale bar indicates 400 μm. HN3: an antibody specific for the core protein of GPC3. The open wound area at 0 hours was regarded as 100%. Values represent mean ± SD from three replicates. P**<0.01 and P***<0.001 compared to IgG group. (C) Wound healing assay to measure cell migration ability on Hep3B cells treated with different concentrations of HS20 for 24 hours. The open wound area at 0 hours of HS20 treatment was regarded as 100%. Values represent mean ± SD from three replicates. P*<0.05 and P**<0.01. (D) Time course of wound healing assays on Hep3B cells treated with 50 μg/mL HS20. The open wound area at 0 hours of HS20 treatment was regarded as 100%. Values represent mean ± SD from three replicates. P*<0.05 and P**<0.01. (E) Hep3B scr cells and Hep3B GPC3-knockdown cells (sh-2) were treated with 100 μg/mL HS20 or human IgG, and then the wound healing assay was performed. In each group, the open wound area of HS20 treatment was compared to that of IgG treatment and is shown as the fold of the open area. Scale bar indicates 400 μm. Values are mean ± SD from three replicates. P**<0.01. (F) Trans-well assay to examine cell motility in Hep3B, Huh-7, and SK-hep1 cells pre-treated with 50 μg/mL IgG or HS20. Scale bar indicates 50 μm.

To determine whether blocked HS chains on GPC3 could affect cell migration, we treated HCC cells with HS20, a human monoclonal antibody that recognizes the HS chains on GPC3 [9], and then analyzed cell migration and motility. HS20 reduced Hep3B migration but had no effect on SK-hep1, a GPC3-negative cell line (Fig 2A). Interestingly, an antibody (HN3) that recognizes the core protein of GPC3 [11] did not inhibit Hep3B cell migration (Fig 2B), suggesting that the HS chains of GPC3 play potentially critical roles for HCC cell migration. HS20-induced inhibition occurred in a dose-dependent manner. With HS20 treatment, inhibition could be observed at a concentration as low as 10 μg/mL (Fig 2C). With 50 μg/mL HS20 treatment, cell mobility was significantly reduced after 24 hours. The wound closure efficiency of HS20-treated Hep3B cells showed more than a 30% decrease compared to that of the control group (Fig 2D). To evaluate whether GPC3 mediates this HS20-induced inhibition, we examined cell migration ability in GPC3 knockdown cells. Hep3B and Hep3B GPC3 knockdown cells were treated with HS20 in a wound healing assay. After 30 hours, the migration rate of control cells was inhibited by HS20. However, the migration of GPC3 knockdown cells was not significantly reduced (Fig 2E). HS20 also showed inhibitory effects on Hep3B and Huh-7 cell motility, whereas HS20 did not influence the cell motility of SK-hep1 cells (Fig 2F). This data indicates that HS20 inhibits HCC cell migration and motility by neutralizing the function of HS chains on GPC3.

### HS20 inhibited HGF-induced cell migration and motility in HCC cells

**Fig 3:**
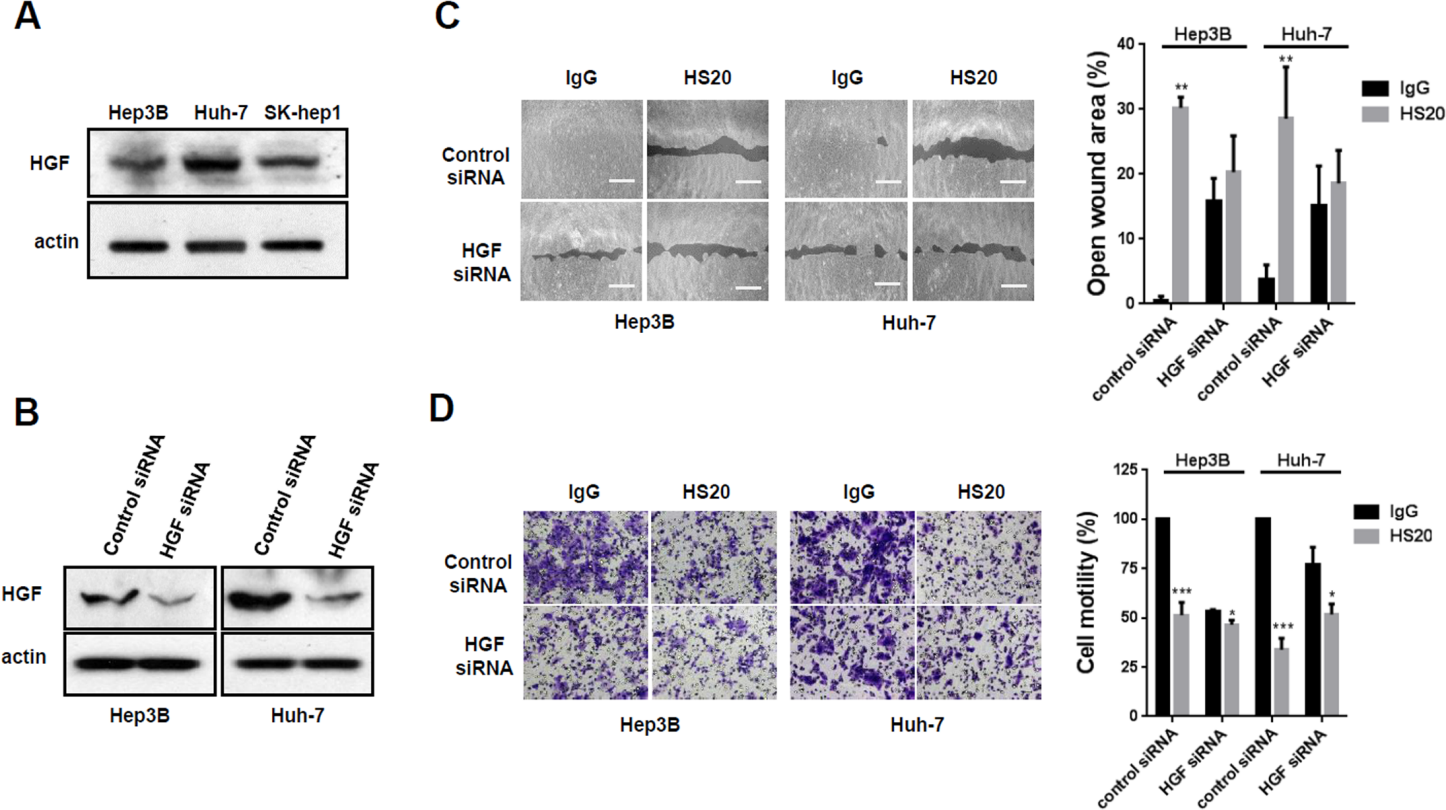
HGF knockdown reduced the inhibitory effect of HS20 in HCC cells. (A)Western blots to examine the expression of HGF in Hep3B, Huh-7 and SK-hep1 cells. (B) Western blots to examine the knockdown efficiency of HGF. (C) Hep3B cells and Huh-7 cells with HGF knockdown were treated with 50 μg/mL human IgG or HS20. Cell migration ability was then measured with a wound healing assay. The open wound area at 0 hours was regarded as 100%. Scale bar indicates 400 μm. Values represent mean ± SD from three replicates. P**<0.01 compare to IgG group. (D) Hep3B cells and Huh-7 cells with HGF knockdown were treated with 50 μg/mL human IgG or HS20. A Trans-well assay was performed to examine cell motility. The OD_590nm_ value of control siRNA group with IgG treatment was set up as 100%. Scale bar indicates 50 μm. Values are mean ± SD from three replicates. P*<0.1 and P***<0.01 compared to the IgG group.

To investigate the underlying mechanism of how HS20 regulates cell migration and motility in HCC cells, we initially detected the effect of canonical and non-canonical Wnt signaling on HCC cell motility. Hep3B cells were treated with Wnt3a- or Wnt5a-conditioned media, but none of them had a significant effect on cell migration (data not shown). This observation suggests that GPC3 may coordinate with other signaling to regulate cell movement. Several studies report that glypican-1 (GPC1) and glypican-4 (GPC4) are involved in HGF-dependent signaling [29,30]. Therefore, we detected whether HGF is expressed in HCC cells. As shown in Fig 3A, Hep3B, Huh-7, and SK-hep1 cells all expressed endogenous HGF. We knocked down HGF with siRNA; at least 70% of HGF expression was reduced after transfection (Fig 3B). The HGF knockdown cells had a slower cell migration rate. When treated with HS20, cell migration of HGF knockdown cells was not significantly inhibited (Fig 3C). Similarly, HGF knockdown cells exhibited reduced cell motility. HS20 slightly inhibited cell motility in HGF knockdown cells but the inhibition was significantly less than that of control cells (Fig 3D). All of these observations indicated that HGF regulated HCC cell migration and motility.

**Fig 4:**
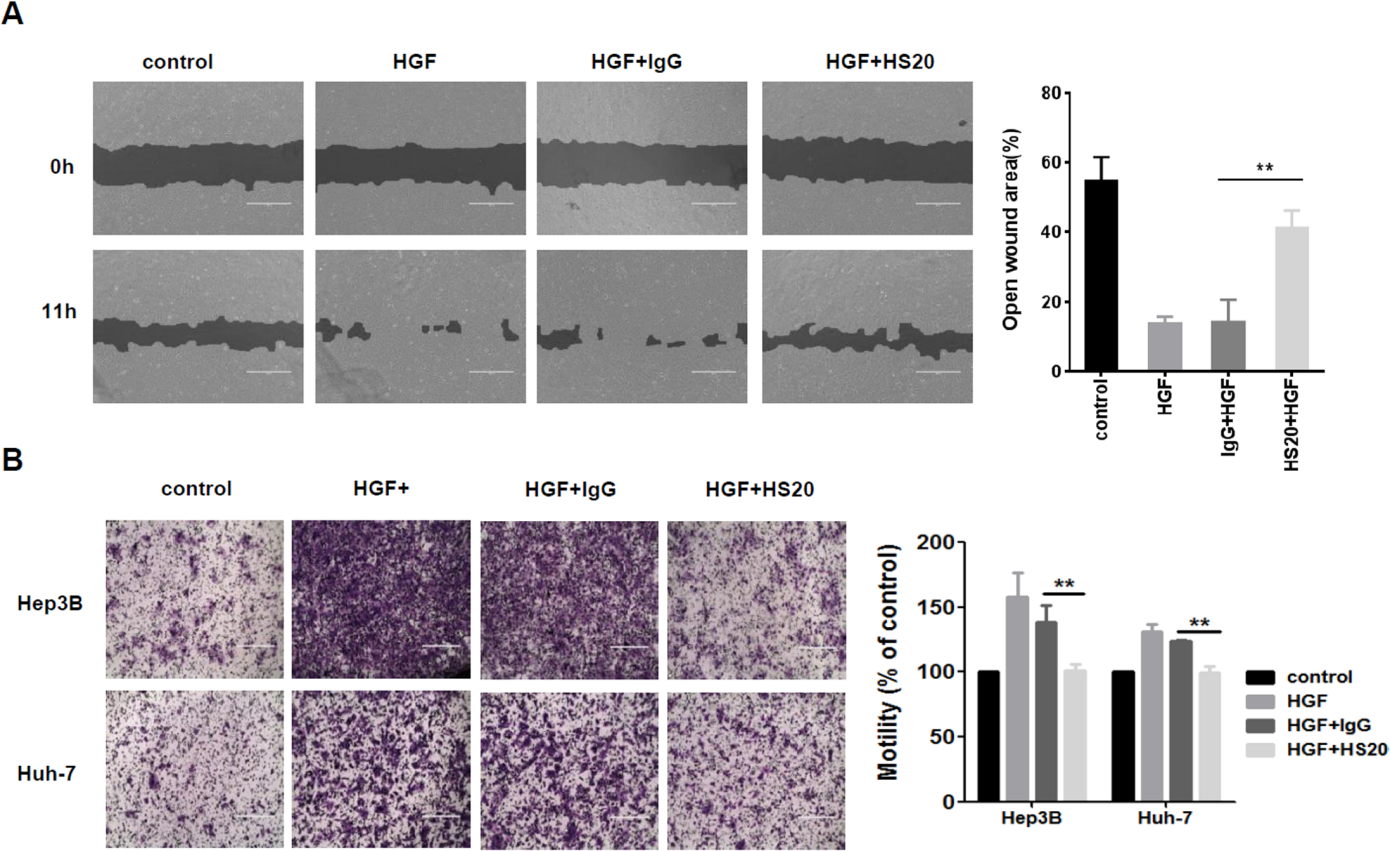
HS20 inhibited HGF-mediated cell migration and motility. (A) Hep3B cells were pre-treated with 50 μg/mL HS20 or human IgG for 30min before adding 50ng/mL HGF. A wound healing assay was performed to detect cell migration ability. Scale bar indicates 400 μm. The open wound area at 0 hours was regarded as 100%. Values represent mean ± SD from three replicates. P**<0.01. (B) Trans-well assay to detect cell motility. Hep3B and Huh-7 cells were seeded into upper wells alone or with 50 μg/mL HS20 or human IgG for HGF chemotaxis. Scale bar indicates 400 μm. The OD_590nm_ value of control group was set up as 100%. Values represent mean ± SD from three replicates. P**<0.01.

Moreover, we treated the cells with purified recombinant HGF and then examined cell migration and motility. We found that HGF stimulation caused dramatically faster cell migration and motility, and when we pre-treated cells with HS20, the increase in cell migration was blocked (Fig 4A). In a cell motility assay, HS20 abolished HGF-driven cell motility in both Hep3B and Huh-7 cells, whereas cells treated with a control antibody did not exhibit significant changes (Fig 4B). These observations indicated that, by neutralizing the function of HS chains on GPC3, HS20 blocked HGF-mediated cell migration and motility of HCC cells.

### The HS chains of GPC3 were involved in HGF/Met activation

**Fig 5:**
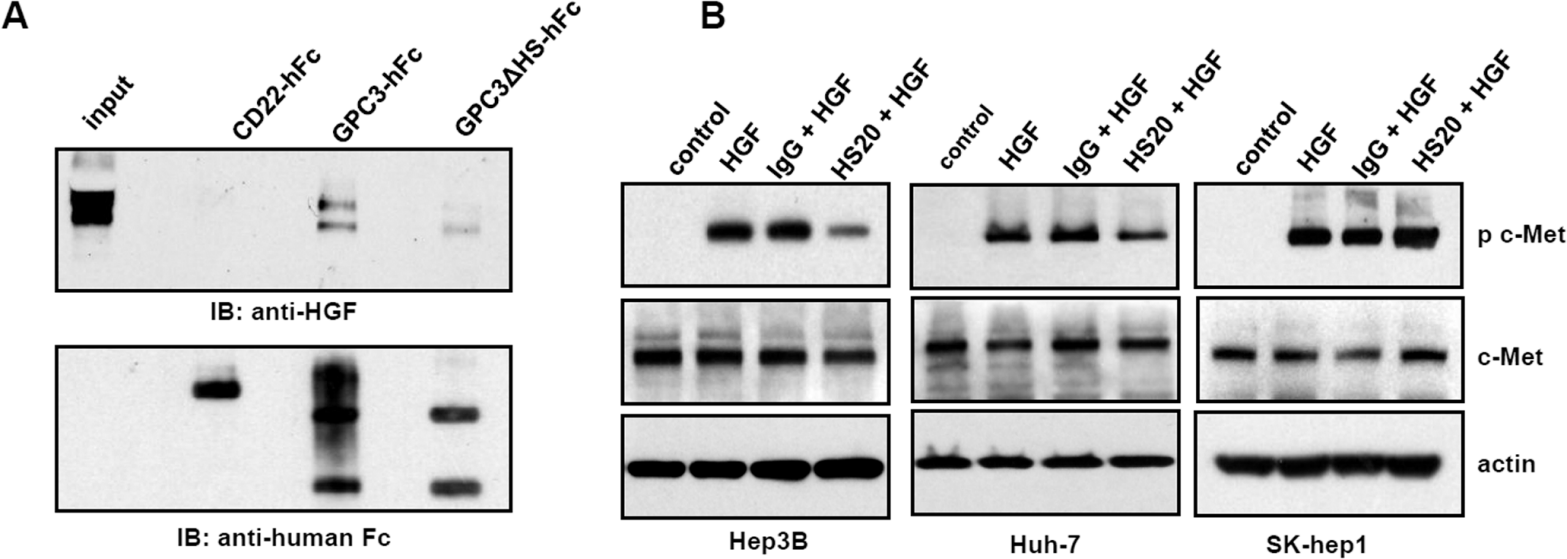
The HS chains of GPC3 were involved in HGF binding and HGF/Met activation. (A) Pull down assay to detect interaction between GPC3-hFc or GPC3ΔHS-hFc and HGF. CD22-hFc was used as an irrelevant protein control. (B) Cells were pre-incubated with 50 μg/mL HS20 or human IgG for 30min and then 50 ng/mL HGF were added to treat cells for 10min. A Western blot was performed to detect the expression of total c-Met and phosphorylated c-Met.

To evaluate whether the HS chains of GPC3 play an important role in HGF activation, we examined the interaction of GPC3 and HGF. We incubated purified GPC3 or mutant GPC3 without HS chains (GPC3ΔHS) with recombinant HGF to perform a pull down assay. As shown in Fig 5A, GPC3 interacted with HGF but not with the control protein CD22. Compared to GPC3, GPC3ΔHS showed a greater reduction in binding with HGF, indicating that the HS chains contribute to GPC3 and HGF interaction. Moreover, we pre-incubated HCC cells with HS20 before HGF treatment and found that HS20-treated HCC cells showed decreased levels of phosphorylated c-Met compared to control IgG-treated groups in Hep3B cells and Huh-7 cells, but not GPC3-negative SK-hep1 cells (Fig 5B). Altogether, these observations suggest that the HS chains on GPC3 are involved in HGF/Met activation in liver cancer cells.

### Targeting the HS chains of GPC3 suppressed *in vitro* HCC spheroid formation and liver tumor growth in mice

**Fig 6:**
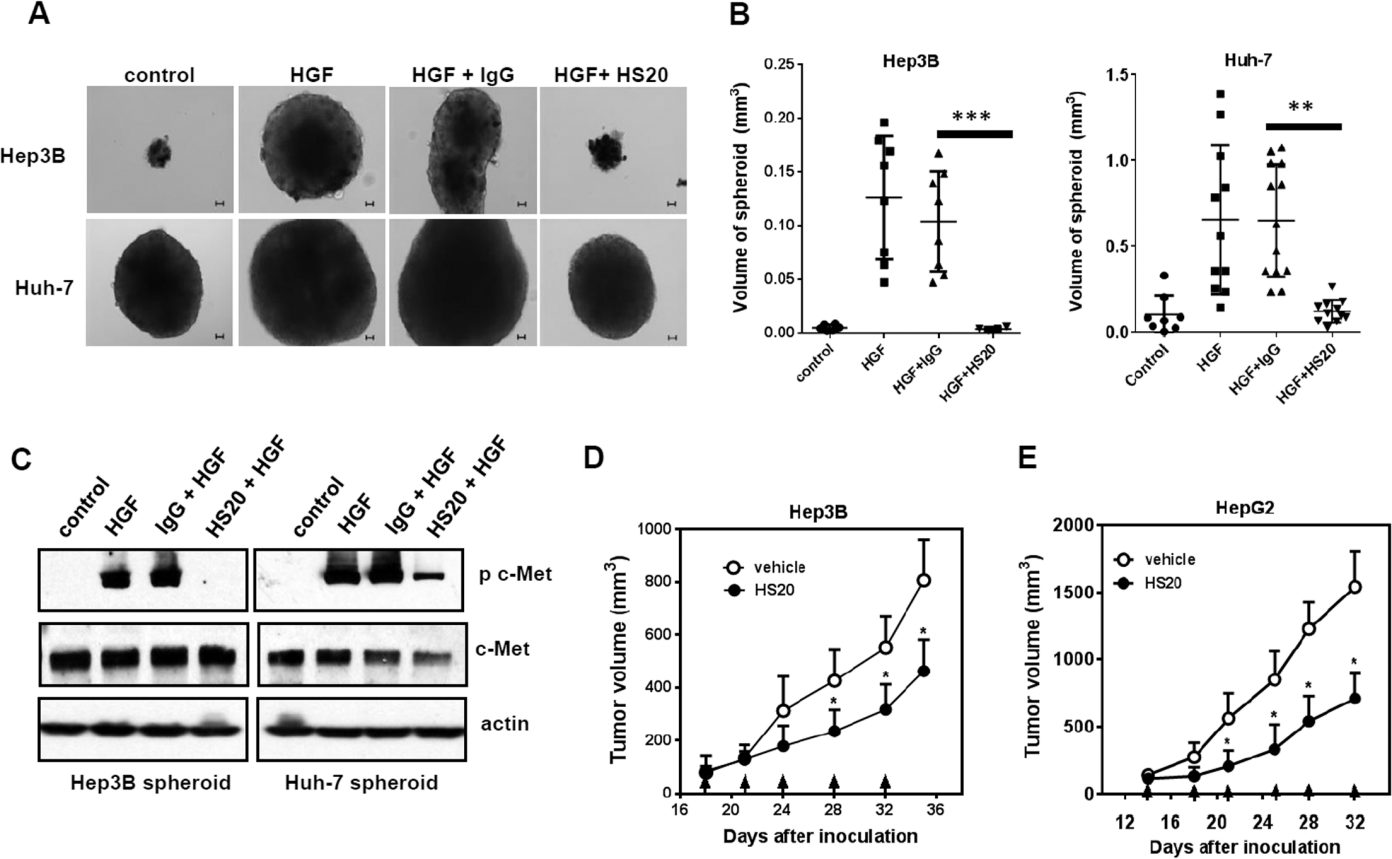
HS20 inhibited HGF-induced tumor spheroid formation. (A) Representative photographs of Hep3B and Huh-7 spheroid. Hep3B and Huh-7 cells (5000 cell/well in 6-well low attachment plate) were treated with 50ng/ml HGF alone or co-cultured with 50 μg/mL HS20 for 20 days. Human IgG was used as negative control. Scale bar indicates 50 μm. (B) The spheroid volume in each group described in (A). Each dot represents a spheroid. P**<0.01 and P***<0.001. (C) Western blot to detect the expression of total c-Met and phosphorylated c-Met in spheroid. Hep3B cells and Huh-7 cells (5000 cell/well in a 6-well low attachment plate) were co-cultured with 50ng/ml HGF and 50 μg/mL HS20 for 20 days in a low attachment plate. Human IgG was used as a negative control. (D) BALB/c nu/nu mice were subcutaneously inoculated with 10x10^6^ Hep3B cells. When tumors reached an average volume of 100 mm^3^, mice were grouped and intravenously administered 25mg/kg HS20 twice a week. Values are mean ± SE from different mice. P*<0.05. n = 4 for each group. PBS was used as vehicle. Arrows indicate antibody injection. (E) BALB/c nu/nu mice were subcutaneously inoculated with 5x10^6^ HepG2 cells. When tumors reached an average volume of 100 mm^3^, mice were grouped and intravenously administered 20mg/kg HS20 twice a week. Values are mean ± SE from different mice. P*<0.05. n = 5 for each group. PBS was used as vehicle. Arrows indicate antibody injection.

To further evaluate the blocking effect of HS20 on HGF-dependent cell-cell interaction, we performeda tumor spheroid formation assay. Solid tumors normally grow in a three-dimensional (3D) conformation that has an uneven distribution of oxygen and nutrients, causing responses different from those of two-dimensional (2D) cultured cells [31]. Cells were first seeded into low attachment plates, and then we tested the effect of HS20 on spheroid formation. After culturing for 20 days, we found that the wells treated with HGF formed spheroids with a significantly larger size (20-fold bigger for Hep3B cells and 4-fold bigger for Huh-7 cells). This HGF-driven spheroid formation was inhibited in the presence of HS20 but not with the control IgG (Fig 6A and 6B). HS20-treated spheroids had less phosphorylated c-Met, indicating that HS20 maintained the inhibitory effect on HGF activation in a 3D-tumor environment (Fig 6C).

To test the efficacy of the HS20 antibody *in vivo*, we subcutaneously inoculated nude mice with Hep3B cells and treated these tumors with HS20 twice a week. After three injections, HS20 treatment inhibited Hep3B tumor growth in mice (Fig 6D). This result was consistent with our *in vitro* observation that HS20 inhibited the spheroid formation of HCC cells. Since we previously reported that HS20 could reduce HCC tumor growth by inhibiting Wnt signaling, both the Wnt and HGF signaling pathways could play a role in HS20’s inhibitory effect. To exclude Wnt signaling, we chose HepG2, a GPC3-positive hepatoblastoma cell line expressing a constitutively activated β-catenin [32], to establish a xenograft model and evaluate the anti-tumor activity of the HS20 antibody. After two injections, we found that the tumors in the treatment group grew more slowly than those in the vehicle control group (Fig 6E). The data supported that HS20 could inhibit GPC3-positive liver tumor growth *in vivo* via signaling pathways other than the canonical Wnt/β-catenin pathway.

## Discussion

HSPGs play pivotal roles in tumorigenesis, tumor progression, and metastasis [5]. These processes can be mediated by interactions with the HS chains of HSPGs. The HS chains serve as co-receptors for growth factors and facilitate ECM-growth factor interaction [5,33]. In the present study, we found that the HS chains of GPC3 were involved in HCC cell migration via coordination with HGF signaling. Our findings suggest the role of HS in cell motility and provide evidence of the inhibition of tumor pathogenesis by targeting the HS domain of HSPGs.

The emerging role of HSPG in tumor progression supports HS-based treatment for cancer therapies. One such strategy involves the heparanase inhibitor PI-88, which is a highly sulfated oligosaccharide mixture [34]. PI-88 can inhibit angiogenesis and tumor growth by preventing FGF and VEGF receptor-HS interaction, and it is currently in a phase III clinical trial for HCC after surgical resection [35–37]. PG545, an analog of PI-88, has been selected as the leading clinical candidate and is currently in a phase I clinical trial [38]. Delteparin, a low molecular weight non-anticoagulant heparin, also shows promising efficacy in the treatment of small cell lung cancer [39]. These studies indicate that targeting HS may be a feasible option for cancer therapy. However, HS mimics alone may not provide effective anti-tumor treatment due to their limited specificity and potential side effects. Antibody therapy could represent a promising approach for HCC therapy given its high specificity to the tumor antigen. In addition to affecting HCC cells, HS20 also blocks C-met activation in HepG2, a hepatoblastoma cell line with GPC3 expression. This provides the potential application of HS20 in different liver malignancies.

GPC3 participates in HCC pathogenesis via multiple signaling mechanisms. Our previous study shows that HS20 blocks the interaction of GPC3 and Wnt3a, and subsequently inhibits the activation of canonical Wnt signaling. After we treated the mice bearing Hep3B xenografts with HS20, tumor growth was reduced by around 50%. Moreover, HS20 also showed an inhibitory effect on HepG2 tumor growth; after GPC3 was knocked down, tumor growth was no longer inhibited [9]. These results showed that the HS20 antibody had significant anti-tumor activity against HCC and other GPC3-positive liver tumors in mice. Interestingly, HepG2 cells have constitutively activated β-catenin signaling [32]. Therefore, the inhibitory effect of HS20 on HepG2 tumor growth is unlikely to be attributed to the inhibition of Wnt signaling. In our current study, we show that GPC3 coordinates with HGF signaling in liver malignancy through its HS chains as well. The HS chains of GPC3 affect HGF binding but do not seem necessary for Wnt binding [10]. This difference suggests that the HS chains of GPC3 are more relevant to the function of HGF. However, once cells are treated with HS20, both Met and Wnt activation are blocked efficiently. HS20 inhibits Wnt3a-dependent cell proliferation and HGF-induced cell migration, motility, and spheroid formation in HCC cells. Due to the limited understanding of HS structure, it is still a challenge to identify the specific binding motif on HS for either HGF or Wnt. It is possible that the function of HS chains relies more on the specific surface microenvironment of tumor cells. The HS chains could preferentially bind to certain types of factors or recruit whatever molecules carry the opposite charge, increasing the surface concentration of these factors and facilitating their receptor binding. In this case, Wnt and HGF may not be the only pathways blocked by HS20. Since most of these growth factors and their receptors (including HGF and Met) are usually ubiquitously expressed, normal tissues could also be affected if we directly target them. To avoid this, targeting tumor-specific HSPGs to indirectly disturb these signaling pathways with an HS-specific antibody like HS20 would be a more desirable strategy.

Many early studies on HSPGs focus on the composition, biosynthesis, and binding properties of the HS chains [40]. Due to the high diversity of HS fine structures and their biological functions, studies in this field are limited by the lack of specific methodology to distinguish certain types of HS with others. HS20 recognizes a conserved HS structure of glypican proteins [41]. In this regard, HS20 may be useful for multiple tumor types by targeting different glypicans, such as GPC1 in pancreatic cancer [42,43], GPC2 in pre-B cell ALL [44], and GPC5 in rhabdomyosarcoma [45,46].

In conclusion, we reported the role that the HS chains of GPC3 play in liver cancer cell migration and motility. This work may support a rationale for neutralizing HS in tumors for cancer therapies.
